# Potential Co-Factor Role of Tobacco Specific Nitrosamine Exposures in the Pathogenesis of Fetal Alcohol Spectrum Disorder

**DOI:** 10.17140/GOROJ-2-125

**Published:** 2016-03-15

**Authors:** Valerie Zabala, Elizabeth Silbermann, Edward Re, Tomas Andreani, Ming Tong, Teresa Ramirez, Fusun Gundogan, Suzanne M. de la Monte

**Affiliations:** 1 Molecular Pharmacology and Physiology Graduate Program, Brown University, Providence, RI, USA; 2 Department of Neurology, Washington University, St. Louis, MO, USA; 3 Alpert Medical School of Brown University, Providence, RI, USA; 4 Graduate Program in Neuroscience, Northwestern University, Chicago, IL, USA; 5 Liver Research Center, Division of Gastroenterology and Department of Medicine, Rhode Island Hospital and the Alpert Medical School of Brown University, Providence, RI, USA; 6 NIAAA, Rockville, MD, USA; 7 Department of Pathology, Women and Infants Hospital of Rhode Island, Alpert Medical School of Brown University, Providence, RI, USA; 8 Departments of Neurology, Neurosurgery, and Pathology, Rhode Island Hospital and the Alpert Medical School of Brown University, Providence, RI, USA

**Keywords:** Insulin resistance, Fetal alcohol spectrum disorder, Tobacco, Cerebellum, NNK, Nitrosamine

## Abstract

**Background:**

Cerebellar developmental abnormalities in Fetal Alcohol Spectrum Disorder (FASD) are linked to impairments in insulin signaling. However, co-morbid alcohol and tobacco abuses during pregnancy are common. Since smoking leads to tobacco specific Nitrosamine (NNK) exposures which have been shown to cause brain insulin resistance, we hypothesized that neurodevelopmental abnormalities in FASD could be mediated by ethanol and/or NNK.

**Methods:**

Long Evans rat pups were intraperitoneal (IP) administered ethanol (2 g/kg) on postnatal days (P) 2, 4, 6 and/or NNK (2 mg/kg) on P3, P5, and P7 to simulate third trimester human exposures. The Cerebellar function, histology, insulin and Insulin-like Growth Factor (IGF) signaling, and neuroglial protein expression were assessed.

**Results:**

Ethanol, NNK and ethanol+NNK groups had significant impairments in motor function (rotarod tests), abnormalities in cerebellar structure (Purkinje cell loss, simplification and irregularity of folia, and altered white matter), signaling through the insulin and IGF-1 receptors, IRS-1, Akt and GSK-3β, and reduced expression of several important neuroglial proteins. Despite similar functional effects, the mechanisms and severity of NNK and ethanol+NNK induced alterations in cerebellar protein expression differed from those of ethanol.

**Conclusions:**

Ethanol and NNK exert independent but overlapping adverse effects on cerebellar development, function, insulin signaling through cell survival, plasticity, metabolic pathways, and neuroglial protein expression. The results support the hypothesis that tobacco smoke exposure can serve as a co-factor mediating long-term effects on brain structure and function in FASD.

## INTRODUCTION

Alcohol abuse during pregnancy causes Fetal Alcohol Spectrum Disorder (FASD) which leads to long-term neurodevelopmental deficits.^1,2^ Ethanol mediates its adverse effects on the immature brain by inhibiting insulin and Insulin-like Growth Factor (IGF) signaling at multiple points within the cascade, beginning with ligand-receptor binding.^3^ Consequently, ethanol inhibits: 1) insulin and IGF-1 Receptor Tyrosine Kinases (RTKs); 2) Insulin Receptor Substrate (IRS) signaling^4–6^; 3) Phosphotidyl-Inositol-3-Kinase (PI3K) and Akt activation; 4) Akt suppression of Glycogen Synthase Kinase-3β (GSK-3β)^3,5–8^; and 5) inactivation of phosphatases that negatively regulate RTKs, i.e. PTP-1b or PI3K, i.e. PTEN.^6,8,9^ These effects compromise growth, survival, metabolism, neuronal migration, and plasticity during development.^10–15^ In addition, acetaldehyde build-up from incomplete alcohol metabolism causes neurotoxic injury due to oxidative stress, DNA damage, mitochondrial dysfunction, and probably adduct formation in brain.^16–21^ Therefore, ethanol inhibition of insulin/IGF signaling combined with acetaldehyde-mediated oxidative injury could be responsible for many of the structural and functional Central Nervous System (CNS) abnormalities in FASD, including motor impairments, cerebellar hypoplasia, and neuronal migration disorders.

Although ethanol exposure is sufficient to cause FASD, in reality, heavy alcohol abusers are often smokers^22^ and both heavy drinking and cigarette smoking adversely affect neurocognitive function.^23^ Review of an outpatient substance abuse treatment center database for pregnant women revealed that from 2010 to 2013, 74% of the pregnant alcohol users (N=57) smoked cigarettes compared with 42% of controls (N=31) (P=0.0053).^24^ Despite strong evidence linking cigarette smoking during pregnancy to impaired fetal growth and development, and neurocognitive function,^25^ the mechanisms are not well understood. Given the frequency of overlapping exposures and their known independent adverse effects on development, further research is needed to determine the degree to which alcohol and tobacco exposures produce additive or synergistic adverse effects on the immature brain.

Nicotine is the main stimulant and addictive substance in tobacco. Nicotine’s half-life is short (1–2 hours), but its chief stable metabolites, cotinine, can cause significant harm. Moreover, tobacco-specific nitrosamines, including 4-(methylnitrosamino)-1-(3-pyridyl)-1-butanone (NNK), N-nitrosonornicotine (NNN), nitrosaminoaldehyde (NNAL), N-nitrosoanatabine (NAT), N-nitrosoanabasine (NAB), iso-NNAL, iso-N-nitrosamino acids (iso-NNAC)^26,27^ are problematic because they are present in tobacco smoke and cause DNA damage and form adducts with proteins, lipids, and nucleic acids.^28–30^ In previous studies, we found that low-dose exposures to N-nitrosodiethylamine, a nitrosamine found in processed and preserved foods, cause sustained deficits in neurobehavioral function with impairments in brain insulin/IGF signaling, increased oxidative stress, and reduced expression of neuroglial genes.^31^ Subsequently, we demonstrated similar independent or additive adverse effects of ethanol and low-dose NNK exposures in adolescent rats,^32,33^ and that low-dose NDEA exposures exacerbate the injurious effects of ethanol on the developing brain.^34^ These findings suggest that further studies concerning additive and interactive effects of ethanol and nitrosamine exposures during development are needed to understand the nature and mechanisms of their long-term effects.

The prevalent co-morbid exposures to alcohol and tobacco during pregnancy prompted us to evaluate neurotoxic and teratogenic effects of tobacco-specific nitrosamine exposures in relation to the effects of alcohol. This study utilized an *in vivo* model of ethanol and/or NNK exposures during the early postnatal period to examine effects on cerebellar function, structure, insulin and IGF-1 signaling through survival, metabolic, and plasticity pathways, and neuroglial protein expression. The findings demonstrate that early postnatal exposures to ethanol, NNK, or ethanol+NNK produce molecular, biochemical, and histopathological effects that correspond with abnormalities in FASD. Therefore, the pathogenesis of sustained neurodevelopmental abnormalities are typically linked to prenatal alcohol exposures may be mediated in part by co-exposures to tobacco smoke.

## METHODS AND MATERIALS

### *In vivo* Model

The models generated were intended to simulate 3^rd^ trimester pregnancy human exposures to alcohol and/or tobacco toxins. Long Evans rat pups were divided into 4 groups and administered 50 μl i.p. injections of: saline vehicle as control; pharmaceutical grade ethanol (2 g/kg in saline); NNK (2 mg/kg in saline); and ethanol+NNK. Ethanol treatments (binge) were administered on postnatal days (P) 2, 4, 6, and 8,^35–37^ and NNK was administered on P3, P5, P7, and P9. All rats survived the treatments and growth trajectories were similar across the groups. All experiments were performed in accordance with protocols approved by Institutional Animal Care and Use Committee at the Lifespan-Rhode Island Hospital, and they conformed to guidelines established by the National Institutes of Health.

### Rotarod Testing

We used rotarod tests to assess cerebellar motor function.^38^ On P16, rats (N=6–8 per group) were administered 10 rotarod trials at incremental speeds up to 10 rpm using a Rota-mex-5 (Columbus Instruments, Columbus, OH, USA), with 10 minutes rest between trials. The latencies to fall were automatically recorded with photocells placed over the rod; however, trials were stopped after 30 seconds to avoid exercise fatigue. Data from trials 1–3 (2–5 rpm), 4–7 (5–7 rpm), and 8–10 (8–10 rpm) were culled and analyzed using the Mann-Whitney test.

### Cerebellar Protein Extraction

On P30, the rats were sacrificed to harvest cerebella for histological and molecular studies. Fresh cerebella were divided in the mid-sagittal plane. One hemisphere was fixed in 10% neutral buffered formalin and embedded in paraffin. Histological sections (5 μm-thick) were stained with Luxol fast blue-Hematoxylin and Eosin (LHE). The other hemisphere was snap frozen and stored at −80 °C. For multiplex and duplex Enzyme-Linked Immunosorbant Assays (ELISAs), fresh frozen cerebellar tissue was homogenized in buffer containing 50 mM Tris (pH 7.5), 150 mM NaCl, 5 mM EDTA (pH 8.0), 50 mM NaF, 0.1% Triton X-100, and protease and phosphatase inhibitors. Supernatants obtained by centrifuging the samples at 14,000 × g for 15 minutes at 4 °C were used in ELISAs. Protein concentrations were measured using the bicinchoninic assay.

### Multiplex ELISAs

Bead-based Total and Phospho Akt Panels were used to examine effects of ethanol, NNK and ethanol+NNK exposures on the expression and phosphorylation of proteins integrally involved in Insulin (IN) and IGF-1 signaling through Akt and Glycogen Synthase Kinase-3β (GSK-3β). The Total Akt multiplex panel measured immunoreactivity to the insulin and IGF-1 receptors, Insulin Receptor Substrate, type 1 (IRS-1), Akt, and GSK-3β. The Phospho-Akt panel measured immunoreactivity to: ^pYpY1162/1163^-IN-R, ^pYpY1135/1136^-IGF-1R, ^pS312^-IRS-1, ^pS473^-Akt, ^pT246^- and ^pS9^-GSK3β. Protein samples (100 μg each) were incubated with antibody-coated beads to capture specific antigens following the manufacturer’s protocol. Biotinylated second epitope antibodies and phycoerythrin-conjugated Streptavidin were reacted with antigen-bound beads, and immunoreactivity was detected and quantified in a MAGPIX (Bio-Rad, Hercules, CA, USA). Data are expressed as Fluorescence Light Units (FLU) corrected for protein concentration.^30,39^

### Duplex ELISAs

Duplex direct-binding ELISAs were used to measure cerebellar protein expression of Hu (neuronal), Myelin-associated glycoprotein-1 (MAG-1; oligodendroglia), Glial fibrillary acidic protein (GFAP; astrocytes), Choline acetyl transferease (ChAT), Acetylcholinesterase (AChE), Glyceraldehyde-3-phosphate dehydrogenase (GAPDH), tau, phospho-tau, ubiquitin, 4-hydroxy-2-nonenal (HNE), and Aspartyl-asparaginyl-β-hydroxylase (ASPH). Quadruplicate 50 μl aliquots containing 2 μg/ml of protein were adsorbed to the bottoms of 96-well MaxiSorp plates by overnight incubation at 4 °C. Non-specific sites were adsorbed with Superblock-TBS. Proteins were reacted with primary antibody (0.1–0.4 μg/ml) overnight at 4 °C. Immunoreactivity was detected with HRP-conjugated secondary antibody and Amplex UltraRed soluble fluorophore. Protein homogenates were subsequently incubated with biotin-conjugated antibodies to large ribosomal protein (RPLPO), and immunoreactivity was detected with streptavidin-conjugated alkaline phosphatase (1:1000) and 4-Methylumbelliferyl phosphate (4-MUP) fluorophore. Fluorescence intensity was measured in SpectraMax M5 microplate reader (Molecular Devices, Sunnyvale, CA, USA) at Ex565nm/Em595nm for Amplex UltraRed, and Ex360nm/Em450nm for 4-MUP. Binding specificity was assessed with control incubations in which the primary or secondary antibody was omitted. The calculated ratios of immunoreactivity corresponding to the specific proteins/RPLPO were used for inter-group comparisons.^39^

## STATISTICS

All assays were performed with 6 or 8 samples per group. Inter-group comparisons were made using two-way ANOVA and the Tukey multiple comparisons post-hoc tests (GraphPad Prism 6, San Diego, CA, USA).

## MATERIALS

Pharmaceutical grade ethanol was used in the *in vivo* experiments. The A85G6 and A85E6 monoclonal antibodies to ASPH were generated to human recombinant protein^40^ and purified over Protein G columns (Healthcare, Piscataway, NJ, USA). Otherwise, antibodies used for duplex ELISAs were purchased from Abcam (Cambridge, MA, USA). RPLPO antibody was from the Proteintech Group Inc (Chicago, IL, USA). ELISA MaxiSorp 96-well plates were purchased from Nunc (Rochester, NY, USA). Horseradish peroxidase (HRP)-conjugated secondary antibody, Amplex Red soluble fluorophore, and the Akt Pathway Total and Phospho panels were purchased from Invitrogen (Carlsbad, CA, USA). HRP-labeled polymer conjugated secondary antibody was purchased from Dako Corp (Carpinteria, CA, USA). The SpectraMax M5 microplate reader was purchased from Molecular Devices Corp. (Sunnyvale, CA, USA). BCA reagents were from Pierce Chemical Corp. (Rockford, IL, USA). All other fine chemicals, including NNK were purchased from CalBiochem (Carlsbad, CA, USA), Pierce (Rockford, IL, USA), or Sigma (St. Louis, MO, USA).

## RESULTS

### Effects of Ethanol and NNK on Motor Function

**Figure 1:**
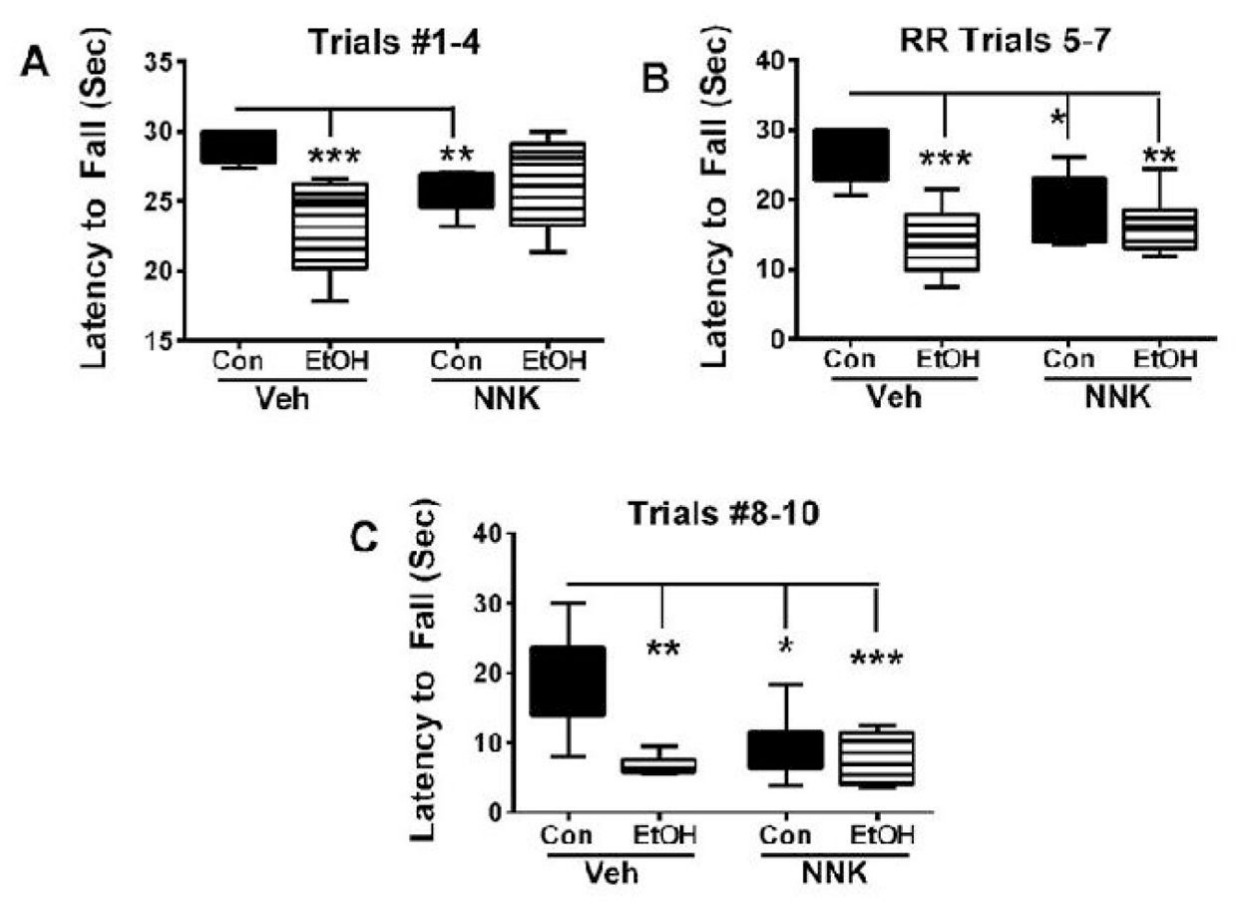
Effects of early postnatal exposures to ethanol, NNK, or ethanol+NNK on motor performance as early adolescence. Long Evans rat pups were treated with i.p. saline (control-vehicle), ethanol (2 g/kg), NNK (2 mg/kg), or ethanol+NNK from postnatal day 2 (P2) through P12. Ethanol was administered on Mondays, Wednesdays, and Fridays, and NNK was administered on Tuesdays, Thursday, and Saturdays. On postnatal day 16, the rats were subjected to 10 trials of rotarod testing with incremental speeds. Data from Trials (A) 1–4, (B) 5–7, and (C) 8–10 were culled and analyzed using the Mann-Whitney test. *p<0.05; **p<0.01; ***p<0.001; ****p<0.0001.

Growth curves were virtually identical for all groups (Supplementary Figure 1). Rotarod latency to fall data were culled for Trials 1–4, 5–7, and 8–10 and analyzed using the Kruskal-Wallis ANOVA and Dunn’s multiple comparison test. Significant inter-group differences were detected for Trials 1–3 (*p*=0.0003), 5–7 (*p*<0.0001), and 8–10 (*p*<0.0001). In Trials 1–4 (least challenging), significant differences from control occurred in the ethanol (*p*<0.001) and NNK (*p*<0.01), but not in the ethanol+NNK group (Figure 1A), but in subsequent more demanding trials, the mean latencies to fall were significantly reduced in all three experimental groups relative control. Furthermore, the effect sizes increased with trial difficulty (Figures 1B, C). Although ethanol and NNK independently impaired motor performance to similar degrees, dual exposure effects were not additive or synergistic.

### Histopathology

**Figure 2:**
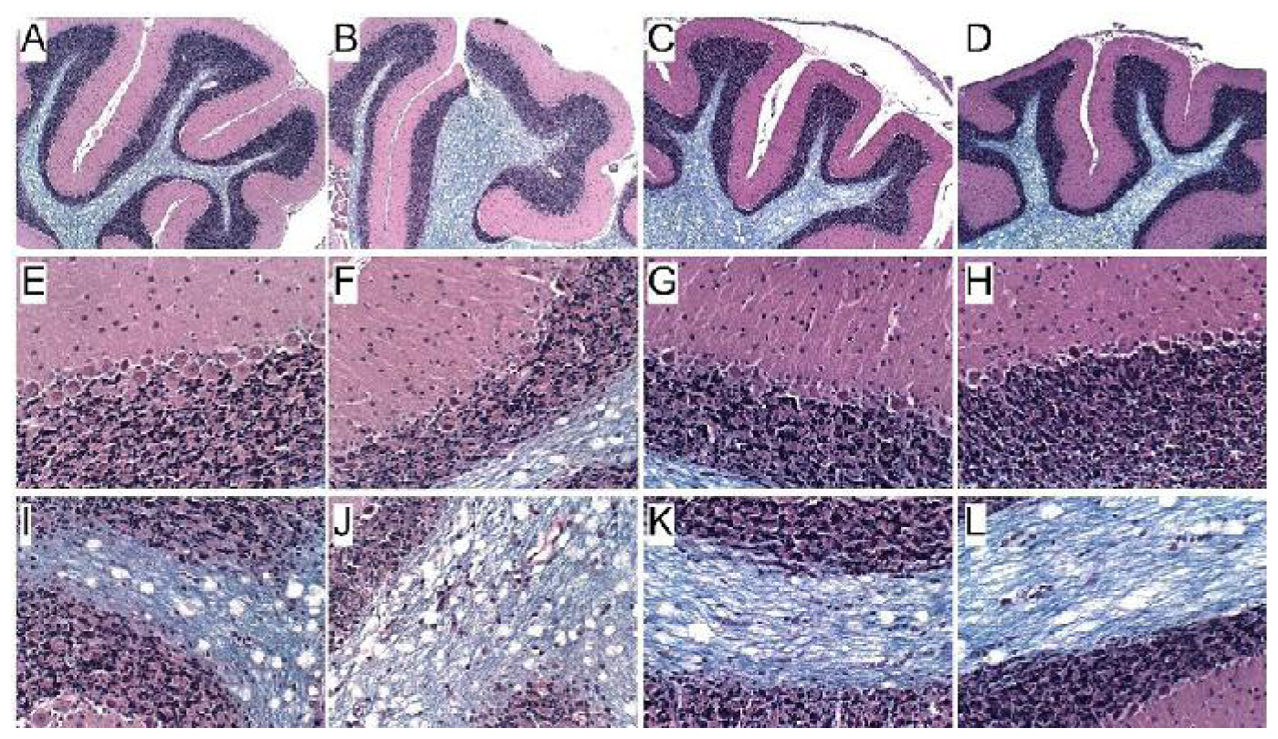
Ethanol and NNK effects of cerebellar structure. Fresh cerebella were hemi-sectioned in the mid-sagittal plane (along the vermis). One half was immersion fixed in formalin and other was snap frozen in a dry ice-methanol bath for later molecular studies. Paraffin-embedded histological sections (8 μm thick) were stained with Luxol fast blue, Hematoxylin and Eosin (LHE). (A, E, I) Vehicle treated control cerebellar cortex showing (A complex foliation (folding), (E) uniform molecular layer and well-populated granule and Purkinje cell layers, and (I) well-delineated and densely stained (blue) white matter cores. (B, F, J) NNK−, (C, G, L) ethanol−, and (D, H, M) ethanol+NNK-exposed cerebella showing relative simplification of folia with reduced secondary sulcation (arrows in A), thinner molecular (m) layers (compare B–D to A), reduced densities of granule cells (gc) and Purkinje cells (P) in Panels F–H versus E, and broader white matter (wm) cores with reduced intensity of myelin staining (compare wm in J–M to I). Original magnifications, A–D, 50x; E–H, 200x; I–M, 100x.

LHE stained histological sections of the cerebellar vermis (mid-sagittal section, anterior superior region-lobules IV-VIII) revealed the characteristic 3-layered cortical architecture with distinct white matter cores in all groups (Figure 2). Control cerebella had relatively uniform thicknesses of the molecular and granule cell layers, a well-populated Purkinje cell layer, and delicately arborized, well-myelinated white matter cores (Figures 2A, E, I). Ethanol exposure altered the patterns of foliation, causing mainly shallow grooving, irregular thickness of the molecular layer, loss of Purkinje cells, and prominent broadening with reduced myelin staining and increased vacuolation of white matter cores (Figures 2B, F, J). NNK treatment resulted in thin simplified (straighter) foliation with sulcal widening, prominent irregularity in granule cell layer thickness within the depths of sulci, atrophy and loss of Purkinje cells, and moderate broadening and tapering of white matter cores compared with the effects of ethanol (Figures 2C, G, K). Ethanol+NNK exposures had similar effects on the cortex and white matter as observed with NNK (Figures 2D, H, L).

### Insulin/IGF-1/IRS-1 Signaling

The multiplex ELISA data corresponding to effects of ethanol, NNK, and ethanol+NNK on signaling proteins, phospho-proteins and relative degrees of phosphorylation within the insulin/IGF-1 pathways were analyzed by two-way ANOVA (Table 1).

### Signaling protein expression

**Figure 3:**
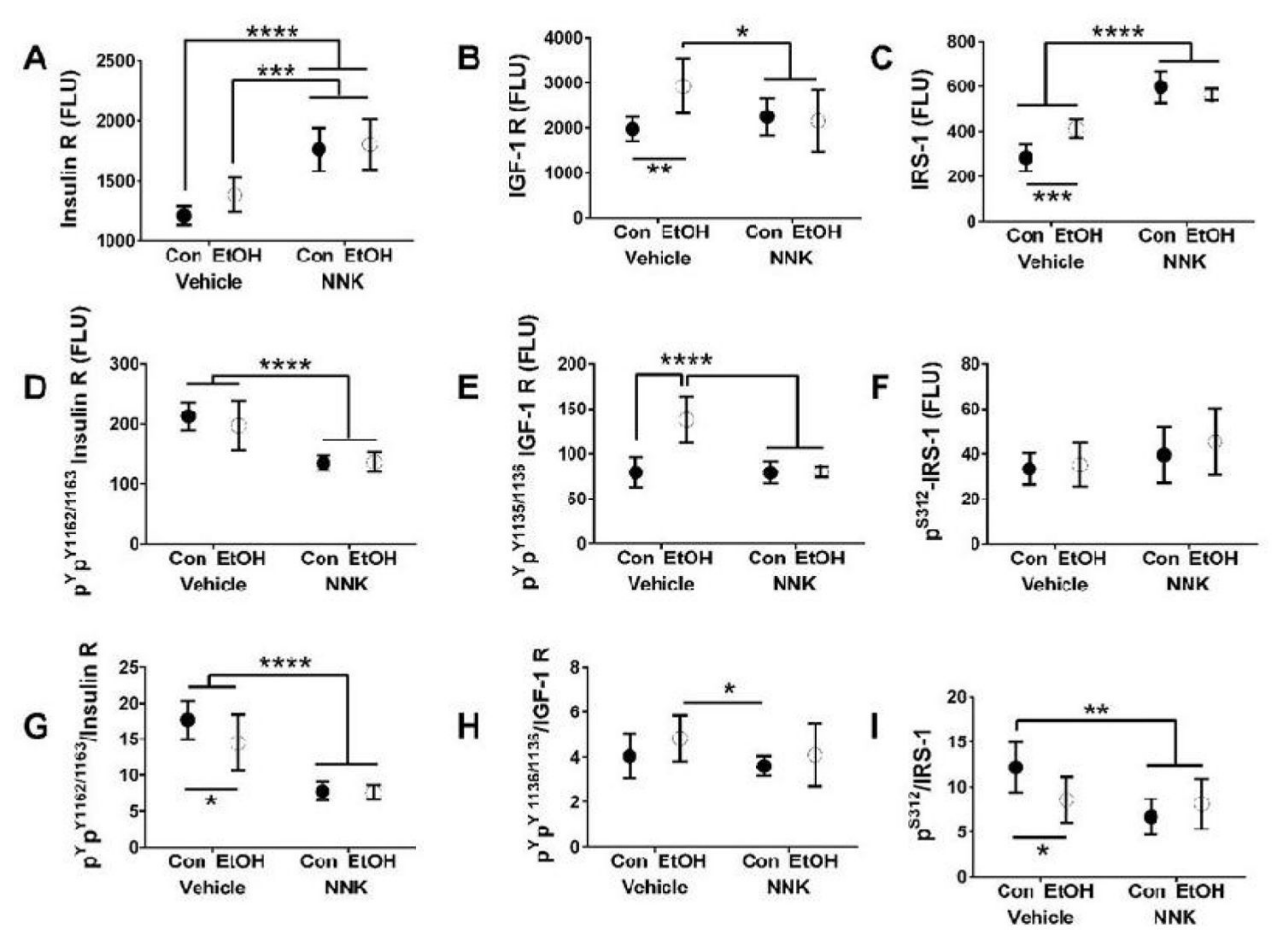
Ethanol and NNK effects on insulin/IGF-1/IRS-1 pathway activation. Cerebellar protein homogenates were used in bead-based multiplex ELISAs to measure immunoreactivity to: (A) insulin receptor (R), (B) IGF-1R, (C) IRS-1, (D) ^pYpY1162/1163^-Insulin-R, (E) ^pYpY1135/1136^-IGF-1R, and (F) ^pS312^-IRS-1. Relative levels of protein phosphorylation are represented by the calculated ratios of (G) ^pYpY1162/1163^-/total Insulin- R, (H) ^pYpY1135/1136^-/total IGF-1R, and (I) ^pS312^-/total IRS-1. Data were analyzed by 2-way ANOVA (Table 1). Graphs depict mean±S.E.M. (N=6 rats/group). 1). Post-hoc Tukey test results are depicted in the graphs (*p<0.05; **p<0.01; ***p<0.001; ****p<0.0001).

**Figure 4:**
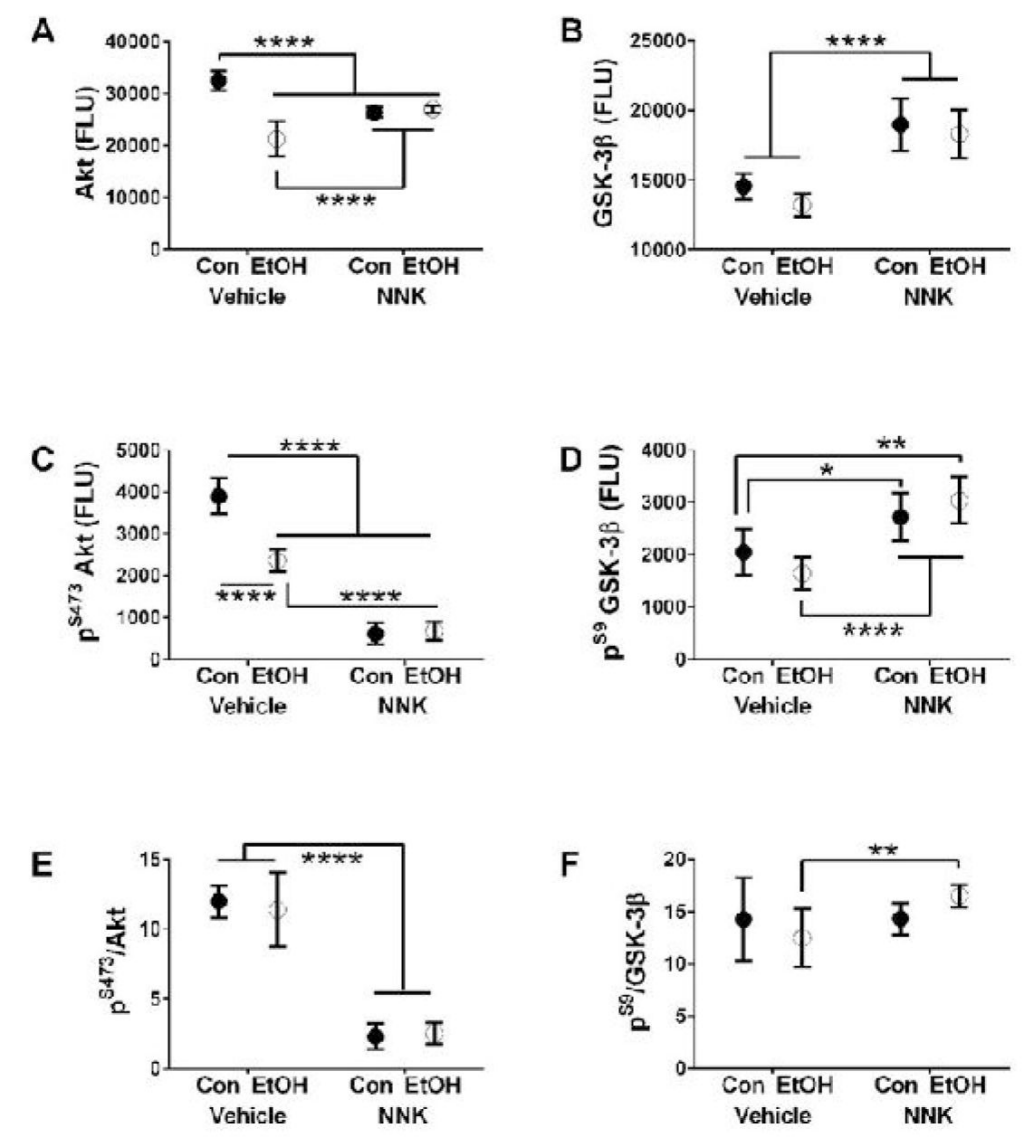
Ethanol and NNK effects on Akt and GSK-3β signaling proteins. Cerebellar protein homogenates were used in bead-based multiplex ELISAs to measure immunoreactivity to: (A) Akt, (B) GSK-3β, (C) ^pS473^ AKT, and (D) ^pS9^-GSK-3β. Relative levels of phosphorylation are represented by the calculated ratios of (E) ^pS473^-/total AKT and (F) ^pS9^-/total GSK-3β. Graphs depict mean ± S.E.M. (N=6 rats/group). Data were analyzed by 2-way ANOVA (Table 1). Post-hoc Tukey test results are depicted in the graphs (^*^p<0.05; ^**^p<0.01; ^****^p<0.0001).

Ethanol had significant effects on IGF-1R, IRS-1, and Akt protein levels, and trend effects (0.05<*p*<0.10) on insulin receptor and GSK-3β. NNK exposures had significant effects on insulin receptor, IRS-1, and GSK-3β expression. Significant ethanol x NNK interactive effects occurred with respect to IGF-1R, IRS-1, and Akt. Post-hoc tests demonstrated that ethanol significantly increased expression of IGF-1R relative to all other groups (Figure 3B), and increased IRS-1 (Figure 3C), but decreased Akt (Figure 4A) relative to control. Effects of NNK and ethanol+NNK were similar in that both types of exposures significantly increased insulin receptor (Figure 3A), IRS-1 (Figure 3C), and GSK-3β (Figure 4B) relative to control and ethanol, and decreased Akt expression relative to control (similar to the effects of ethanol only) (Figure 4A).

### Phospho-protein expression

Ethanol had significant effects on ^pYpY1135/1135^-IGF-1R and ^pS473^-Akt expression. NNK had significant effects on ^pYpY1162/1163^-InsulinR, ^pYpY1135/1135^-IGF-1R, ^pS473^-Akt, and ^pS9^-GSK-3β, and a trend effect on ^S312^-IRS-1 expression. Significant ethanol x NNK interactive effects occurred with respect to ^pYpY1135/1135^-IGF-1R, ^pS473^-Akt, and ^pS9^-GSK-3β overlapping with the effects of NNK- and/or ethanol-only exposures. Graphs and post-hoc tests demonstrated that ethanol significantly increased^pYpY1135/1135^-IGF-1R expression relative to all other groups (Figure 3E), and decreased ^pS473^-Akt relative to control (Figure 4C), paralleling its effects on IGF-1R (Figure 3C) and Akt (Figure 4A). The effects of NNK and ethanol+NNK were similar in that both inhibited^pYpY1162/1163^-InsulinR relative to control (Figure 3D) and^pS473^-Akt relative to control and ethanol (Figure 4C), and increased ^pS9^-GSK-3β relative to control and ethanol-only exposures (Figure 4D). ^S312^-IRS-1 levels were similar across the groups (Figure 3F).

### Relative Protein Phosphorylation

Relative levels of protein phosphorylation were calculated from the ratios of phosphorylated to total protein. An ethanol trend effect occurred with respect to ^pYpY1162/1163^-Insu-linR/InsulinR. Significant NNK effects occurred with respect to ^pYpY1162/1163^- InsulinR/InsulinR, ^S312^-IRS-1/IRS-1, ^pS473^-Akt/Akt, and ^pS9^-GSK-3β/GSK-3β. Significant ethanol x NNK interactive effects were observed for ^S312^-IRS-1/IRS-1 and ^pS9^-GSK-3β/GSK-3β, and a trend effect for ^pYpY1162/1163^-InsulinR/InsulinR. Ethanol reduced expression of ^pYpY1162/1163^-InsulinR/InsulinR (Figure 3G) and ^S312^-IRS-1/IRS-1 (Figure 3I) relative to control. NNK significantly reduced ^pYpY1162/1163^-InsulinR/InsulinR (Figure 3G), ^S312^-IRS-1/IRS-1 (Figure 3I), and ^pS473^-Akt/Akt (Figure 4E) relative to control. Ethanol+NNK exposures significantly reduced^pYpY1162/1163^-InsulinR/InsulinR (Figure 3G), ^S312^-IRS-1/IRS-1 (Figure 3I), and ^pS473^-Akt/Akt (Figure 4E) relative to control, and increased ^pS9^-GSK-3β/GSK-3β (Figure 4F) relative to ethanol-only treatment.

### Neuronal and Glial Protein Expression

Duplex ELISAs were used to measure Hu (neuronal marker), Myelin-associated glycoprotein-1 (MAG-1; mature oligodendrocyte marker), Glial fibrillary acidic protein (GFAP; astrocyte marker), Choline acetyltransferase (ChAT), Acetylcholinesterase (AChE), Glyceraldehyde-3-phosphate dehydrogenase (GAPDH), tau (major neuronal cytoskeletal protein), phospho-tau (phosphorylated on S396 and T205), ubiquitin, 4-hydroxy-nonenal (HNE; lipid peroxidation marker), and Aspartyl-β-hydroxylase (ASPH; protein regulating neuronal migration). Immunoreactivity was normalized to large ribosomal protein (RPLPO). Two-way ANOVAs demonstrated significant ethanol effects on ASPH-A86G6, and a trend effect for HNE (Table 2). Significant NNK effects occurred with respect to MAG-1, GFAP, ChAT, AChE, GAPDH, Tau, pTau, ubiquitin, HNE, ASPH-A85G6, and ASPH-A85E6, but not Hu. Significant ethanol x NNK effects occurred with respect to MAG-1, ChAT, AChE, GAPDH, ubiquitin, HNE, ASPH-A85G6 and ASPH-A85E6.

**Figure 5:**
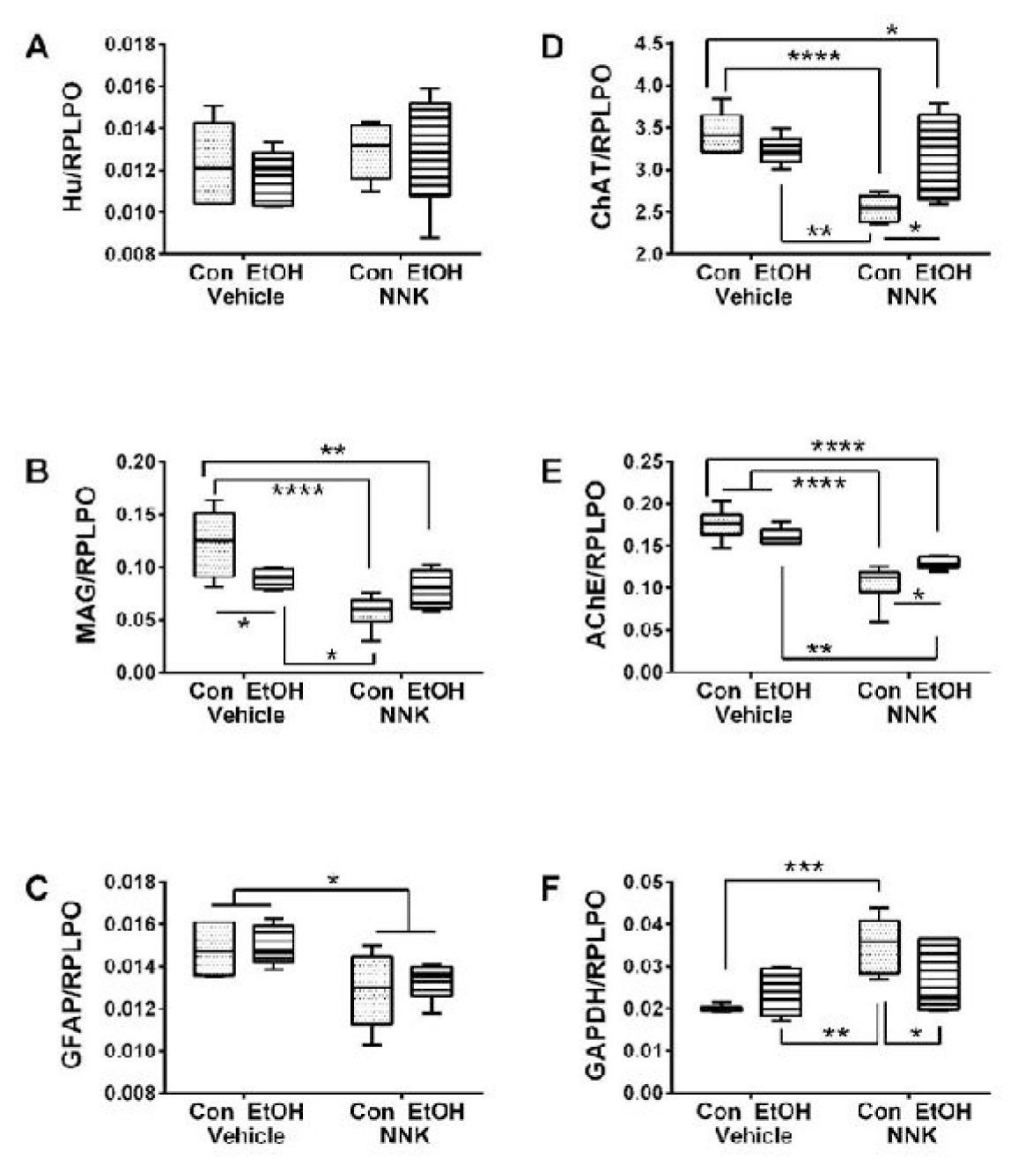
Ethanol and NNK effects on cerebellar neuronal and glial protein expression. Duplex ELISAs were used to measure Immunoreactivity to (A) Hu, (B) myelin-associated glycoprotein 1 (MAG-1), (C) glial fibrillary acidic protein (GFAP), (D) choline acetyltransferase (ChAT), (E) acetylcholinesterase (AChE), and (F) glyceraldehyde-3-phosphate dehydrogenase (GAPDH) with results normalized to RPLPO (control). Data were analyzed by two-way ANOVA (Table 2). Post hoc significance tests determined the specific inter-group differences as shown in the panels. *p<0.05; **p<0.01; ***p<0.001; ****p<0.0001.

**Figure 6:**
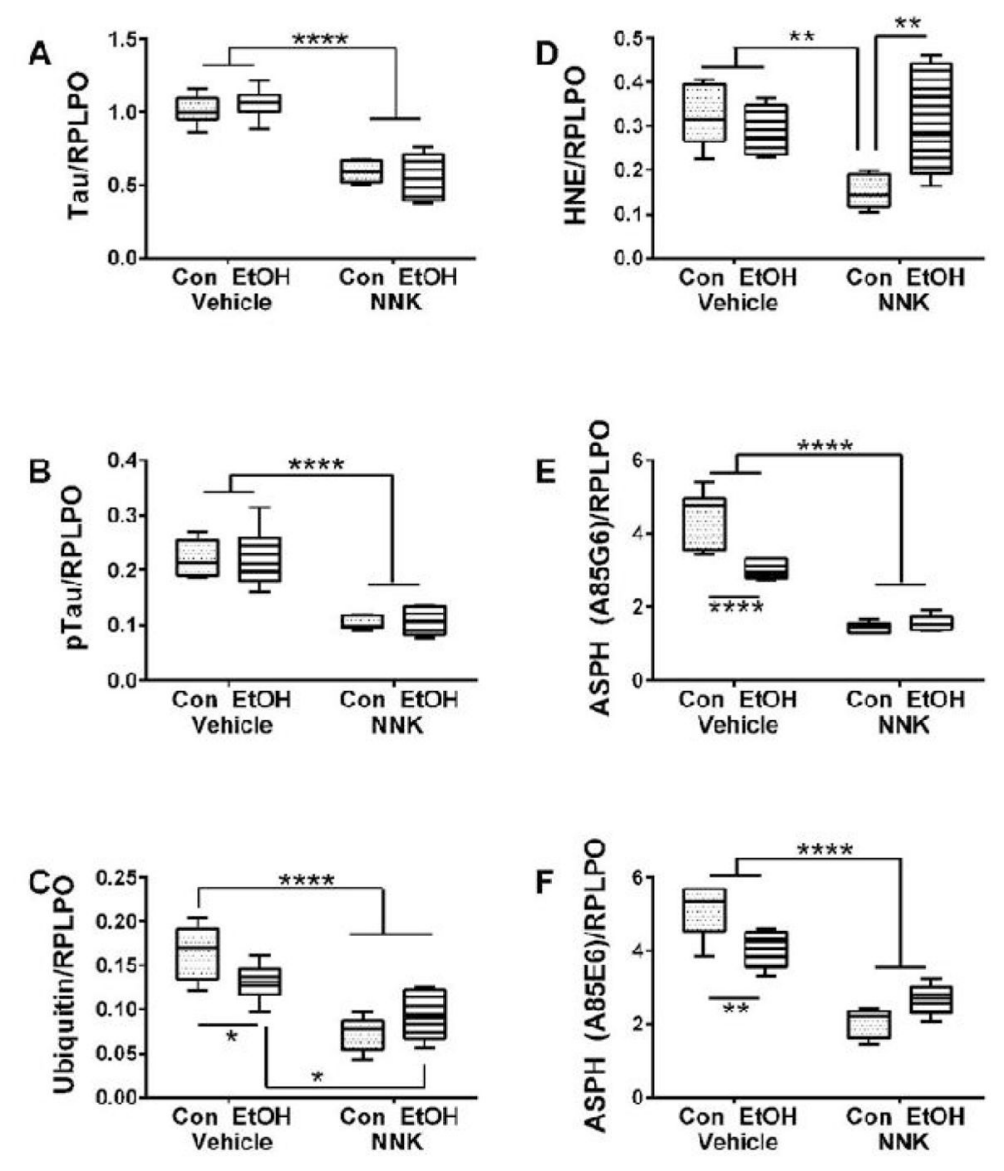
Ethanol and NNK modulation of neuronal and stress protein expression. Cerebellar protein homogenates were used to measure (A) Tau, (B) pTau, (C) ubiquitin, (D) 4-hydroxy-2-nonenal (HNE), (E) aspartate- β-hydroxylase (ASPHA85G6), and (F) ASPH-Humbug (A85E6) immunoreactivity by duplex ELISAs with results was normalized to RPLPO. Inter-group comparisons were made by two-way ANOVA (Table 2) with the post-hoc Tukey multiple comparison test.

Post-hoc tests demonstrated that ethanol significantly reduced expression of MAG-1 (Figure 5B), ubiquitin (Figure 6C), ASPH-A85G6 (Figure 6E), and ASPH-A85E6 (Figure 6F) relative to control. NNK and ethanol+NNK inhibited expression of MAG-1 (Figure 5B), GFAP (Figure 5C), ChAT (Figure 5D), AChE (Figure 5E), Tau (Figure 6A), pTau (Figure 6B), ubiquitin (Figure 6C), ASPH-A85G6 (Figure 6E), and ASPH-A85E6 (Figure 6F) relative to control. In addition, the expression levels of MAG-1, GFAP, ChAT, AChE, Tau, pTau, HNE, ASPH-A85G6 and ASPH-A85E6 were significantly reduced in NNK relative to ethanol-only cerebella. Similarly, the mean expression levels of GFAP, AChE, Tau, pTau, ubiquitin, ASPH-A85G6 and ASPH-A85E6 were significantly reduced the ethanol+NNK relative to the ethanol-only group. Finally, significant differences between NNK and ethanol+NNK were detected for ChAT (Figure 5D), AChE (Figure 5E), GAPDH (Figure 5F), and HNE (Figure 6D), although those responses tended to be normalizing rather than additive or synergistic. Hu was the only protein found to be similarly expressed in all four groups (Figure 5A), and GAPDH was the only protein that was selectively increased by NNK relative to the other groups (Figure 5F).

## DISCUSSION

FASD is caused by prenatal alcohol exposures, either chronic or binge, and associated with a range of neurocognitive and motor deficits. Impairments in motor function are associated with hypoplasia and disordered migration of cerebellar neurons. Mechanistically, alcohol disrupts neuronal insulin and IGF signaling networks needed for neuronal growth, survival, metabolism, migration, plasticity and neurotransmitter function.^1,2^ These adverse effects of ethanol are mediated at all levels of the cascade, from ligand-receptor binding and activation of receptor tyrosine kinases, through downstream Akt and GSK-3β pathways.^3,6,7^

Despite evidence that developmental exposures to ethanol are sufficient to cause FASD, variability in the phenotypic features, including dose-effects, prompted us to consider potential co-factor exposure contributions. The finding of high rates of smoking in women who drank during pregnancy^24^ turned our attention to the concept that tobacco smoke and toxin exposures may contribute to neurodevelopmental abnormalities in FASD. Although tobacco smoke contains hundreds of toxins, tobacco-specific nitrosamines draw interest because, like other nitrosamines, their chronic low-level exposures can cause insulin resistance and neurodegeneration.^32,33^ The present study extends those gains by testing the hypothesis that developmental exposures to low levels of NNK cause cerebellar abnormalities that overlap with or mimic effects of FASD. NNK exposures occur with direct and sidestream (second- hand) tobacco smoke exposures.^41,42^

The third-trimester equivalent exposures were used to target the most vulnerable period of cerebellar development in rats.^43^ Ethanol, NNK, and ethanol+NNK exposures significantly impaired motor performance on rotarod tests and altered cerebellar architecture, including foliation (folding), Purkinje cell density, granule cell layer thickness, white matter structure, and intensity of myelin staining. Therefore, this study demonstrates that low-level NNK exposures during development can impair cerebellar function, but the corresponding effects on cerebellar structure differ. Ethanol-associated alterations in cerebellar foliation (folding and grooving) were associated with neuronal loss in the Purkinje cell layer, whereas NNK and ethanol+NNK caused simplification of cerebellar folia, irregular granule cell layer organization, some neuronal loss in the Purkinje layer, and broadening with pallor of myelin staining in the white matter cores. Therefore, these findings link NNK exposures to sustained functional and structural abnormalities in the cerebellum as occur in FASD. However, the findings also suggest that the mediators of these responses differ for ethanol and NNK.

Multiplex ELISA studies demonstrated that ethanol and NNK impaired signaling through the insulin receptor. However, the ethanol-treated group had increased expression levels of total and ^pYpY1135/1135^-IGF-1R, and all 3 experimental groups had increased levels of IRS-1 protein but decreased levels of ^S312^-IRS-1. Increased expression of IRS-1 enhances downstream signaling through growth and metabolic pathways.^44^ Since ^312^S-phosphorylation of IRS-1 is inhibitory,^45^ its reduced levels *vis-*à*-vis* increased IRS-1 protein expression could represent positive compensatory responses to impaired signaling through the insulin receptor.

The findings that Akt and^S473^-Akt were inhibited by ethanol, NNK, and ethanol+NNK exposures indicates that downstream signaling through growth, metabolic, plasticity, and cell survival pathways were impaired, correlating with cell loss observed in the cerebellar cortex. While these effects of ethanol on the developing brain has been reported,^6,7,46,47^ the new information derived from these studies is that NNK’s neurotoxic effects mechanistically overlap with those of ethanol. Furthermore, the results suggest that the adverse effects of NNK (and smoking) can be worse than ethanol since its inhibitory effects on insulin receptor and Akt signaling were more striking than observed with ethanol. The finding that the dual ethanol and NNK exposures did not produce additive adverse effects on these pathways suggests that corresponds with the broad NNK effects demonstrated by two-way ANOVAs and suggests that maximum pathway dysregulation was produced by the NNK exposures.

Ethanol, NNK and ethanol+NNK significantly altered the expression of several neuroglial proteins in the cerebellum. However, one of the consistent themes was that NNK’s and ethanol+NNK’s inhibitory effects were broader and more striking in severity than those of ethanol. Reductions in MAG-1 expression correlate with the reductions in myelin staining with Luxol fast blue dye and the less sharply delimited architecture of the white matter cores. White matter atrophy, degeneration, and developmental hypotrophy are well-recognized consequences of prenatal ethanol exposure. Recently, we demonstrated similar experimental effects of NNK and tobacco smoke exposures in adolescent or adult brains.^32,48^ The present work provides further evidence that NNK (and probably smoking) impairs white matter myelin development in part by inhibiting expression of mature myelin-associated proteins, including MAG-1. NNK-associated inhibition of GFAP could also indicate that NNK disrupts the structural integrity/scaffolding of the brain, including components needed for blood-brain barrier maintenance and axonal function.

Tau is a key neuronal cytoskeletal protein and tau phosphorylation is critical for translocating the neuronal cytoskeleton from perikarya into neurites, in order to establish and maintain synaptic connections and help axons course through white matter. Since tau expression and phosphorylation are regulated by insulin and IGF-1 signaling and resistance,^49–51^ it is not surprising that both were significantly reduced in NNK- and NNK+ethanol exposed cerebella, given the prominent inhibition of insulin receptor expression and tyrosine phosphorylation in those groups. Similarly, NNK-associated inhibition of ChAT could be attributed to the sustained impairments in insulin signaling because ChAT is regulated by insulin/IGF-1.^7^ Decreased ChAT expression can impair motor function due to reduced cholinergic actions. AChE was also reduced by NNK exposures. Although the mechanism is not well understood, AChE inhibition can be consequential to persistent oxidative stress,^52–55^ as occurs with dysregulation of energy metabolism due to inhibition of Akt signaling. The ethanol and NNK associated reductions in ubiquitin immunoreactivity could reflect deficits in the ubiquitin-proteasome system, as this phenomenon has been reported following chronic ethanol exposure,^56,57^ and in association with various degenerative diseases.^58,59^ Deficits in the ubiquitin-proteasome pathway could lead to increased oxidative and endoplasmic reticulum stress due to activation of the unfolded protein response.^60,61^

For the ASPH measurements, we used two monoclonal antibodies. A85G6 binds to the C-terminal region of ASPH which contains a catalytic domain^40^ required to promote cell motility^62–66^ and neuronal plasticity.^62,63,65,67–70^ A85E6 binds to the N-terminal Humbug-homologous region of ASPH; Humbug has a role in regulating calcium sequestration in the ER.^71^ ASPH is regulated by insulin/IGF-1 signaling through IRS-1 and Akt.^62,67,68^ Inhibition of ASPH perturbs cell motility and adhesion,^63,70,72^ and in the case of FASD, ethanol’s inhibitory effects on ASPH expression correlate with impairments in cerebellar neuronal migration and motor dysfunction.^40,47^ The findings herein demonstrate that developmental exposures to ethanol or NNK significantly inhibit expression of cerebellar ASPH and Humbug proteins, correlating with the persistent alterations in cerebellar architecture and impairments in rotarod motor performance.

In conclusion, ethanol and NNK exposures during development cause structural and functional abnormalities in the cerebellum with associated impairments in signaling through the insulin receptor and Akt. In addition, ethanol and NNK reduced expression of several neuroglial proteins that help maintain the structural and functional integrity of the cerebellum. The effect sizes were generally greater with NNK than ethanol, yet the absence of additive responses suggests that the observed adverse effects of NNK were maximum. Differential responses to ethanol and NNK were evident, indicating that the manners in which they interfere with development also differ. Finally, these studies demonstrate that the CNS abnormalities typically regarded as consequences of prenatal alcohol exposure can be caused by NNK *via* tobacco smoke exposures. Moreover, the findings suggest that the heterogeneity in occurrence and severity of FASD could be due to independent or co-factor effects of tobacco smoke exposures.

## Supplementary Material



## Figures and Tables

**Table 1:** Two-Way ANOVA summary of ethanol and NNK effects on Insulin/IGF-1/Akt signaling networks in the cerebellum-multiplex ELISA results

Protein	Ethanol Effect		NNK Effect		Ethanol x NNK Effect	
	F-Ratio	*P*-Value	F-Ratio	*P*-Value	F-Ratio	*P*-Value
Insulin R	2.944	0.099	59.25	<0.0001	1.092	N.S.
IGF-1 R	4.436	0.046	1.588	N.S.	6.729	0.016
IRS-1	6.107	0.021	140.9	<0.0001	17.05	0.0004
Akt	55.26	<0.0001	0.057	N.S.	68.55	<0.0001
GSK-3β	3.056	0.093	68.91	<0.0001	0.346	N.S.
p-Insulin-R	0.565	N.S.	55.36	<0.0001	0.852	N.S.
p-IGF-1R	23.64	<0.0001	22.43	<0.0001	22.38	<0.0001
p-IRS-1	0.702	N.S.	3.317	0.081	0.202	N.S.
p-Akt	42.57	<0.0001	488.5	<0.0001	51.05	<0.0001
p-GSK-3β	0.075	N.S.	40.47	<0.0001	4.968	0.035
p/T-Insulin R	3.620	0.069	87.61	<0.0001	2.817	0.101
p/T-IGF-1R	2.668	N.S.	2.287	N.S.	0.122	N.S.
p/T-IRS-1	1.320	N.S.	9.585	0.005	6.799	0.015
p/T-Akt	0.137	N.S.	272.9	<0.0001	0.526	N.S.
p/T-GSK-3β	0.062	N.S.	4.723	0.039	4.419	0.046

Cerebellar homogenates were used to measure total and phosphorylated (p) proteins by multiplex bead-based ELISAs. The ratios of phosphorylated/total (p/T) protein were calculated. Data were analyzed by two-way ANOVA. F-ratios reflect variances of the ethanol effects, NNK effects, and ethanol x NNK interactive effects. Italicized P-values represent trend effects. Corresponding graphs with results of post-tests are shown in Figures 3 and 4.

**Table 2:** Two-way ANOVA summary of ethanol and NNK effects on neuronal and glial protein expression in the cerebellum-duplex ELISA results.

Protein	Ethanol Effect		NNK Effect		Ethanol x NNK Effect	
	F-Ratio	*P*-value	F-Ratio	*P*-Value	F-Ratio	*P*-Value
Hu	0.118	N.S.	1.195	N.S.	0.126	N.S.
MAG-1	0.508	N.S.	19.36	0.0003	10.87	0.0036
GFAP	0.307	N.S.	12.63	0.002	0.081	N.S.
ChAT	1.185	N.S.	18.89	0.0003	7.573	0.012
AChE	0.393	N.S.	60.49	<0.0001	8.455	0.009
GAPDH	1.405	N.S.	13.88	0.0013	6.458	0.019
Tau	0.013	N.S.	100.2	<0.0001	0.889	N.S.
pTau	0.016	N.S.	65.19	<0.0001	0.163	N.S.
Ubiquitin	0.525	N.S.	40.38	<0.0001	6.807	0.017
HNE	3.520	0.075	5.982	0.024	8.874	0.007
ASPH-A85G6	14.29	0.001	161.4	<0.0001	20.04	0.0002
ASPH-A85E6	0.949	N.S.	110.8	<0.0001	14.93	0.001

Immunoreactivity was measured by duplex ELISAs with results normalized to the RPLPO control. Data were analyzed by 2-way ANOVA. F-ratios reflect variances for ethanol effects, NNK effects, and ethanol x NNK interactive effects *p*-values represent trend effects. Corresponding graphs with results of post-tests are shown in Figures 5 and 6.
