# Supplementary material for: Potential Co-Factor Role of Tobacco Specific Nitrosamine Exposures in the Pathogenesis of Fetal Alcohol Spectrum Disorder
Source: Gynecol Obstet Res. Author manuscript; Available in PMC 2017 Aug 24. (PMC5570438; doi:10.17140/GOROJ-2-125)
Supplement: Supplementary file 1 [file NIHMS896495-supplement-supplement_1.pdf]

ISSN 2377-1542 Open Journal http://dx.doi.org/10.17140/GOROJ-2-125

notch signaling in cerebellar development and function. *Behav Brain Funct*. 2010; 6: 68. doi: 10.1186/1744-9081-6-68

71. Feriotto G, Finotti A, Breveglieri G, Treves S, Zorzato F, Gambari R. Transcriptional activity and Sp 1/3 transcription factor binding to the P1 promoter sequences of the human AbetaH-J-J locus. *FEBS J*. 2007; 274(17): 4476-4490.

72. Gundogan F, Bedoya A, Gilligan J, Lau E, Mark P, De Paepe ME, et al. siRNA inhibition of aspartyl-asparaginyl beta-hydroxylase expression impairs cell motility, Notch signaling, and fetal growth. *Pathol Res Pract*. 2011; 207(9): 545-553. doi: 10.1016/j.prp.2011.06.001

Supplementary Data

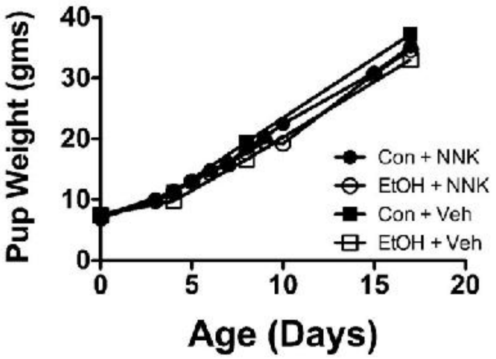

Supplementary Figure 1: Effects of early postnatal ethanol, NNK, and ethanol+NNK exposures on body growth. Long Evans rat pups were divided into 4 groups and administered 50 μl i.p. injections of: saline vehicle as control; pharmaceutical grade ethanol (2g/kg in saline); NNK (2 mg/kg in saline); and ethanol+NNK. Ethanol treatments (binge) were administered on postnatal days (P) 2, 4, 6, and 8, and NNK was administered on P3, P5, P7, and P9. Body weights were obtained at weekly intervals to monitor growth.
